# Supplementary material for: AutoLabs: cognitive multi-agent systems with self-correction for autonomous chemical experimentation
Source: Sci Rep. 2026 Jun 25;16:19554. doi: 10.1038/s41598-026-45593-z (PMC13304172; doi:10.1038/s41598-026-45593-z)
Supplement: Supplementary file 1 — Supplementary Information. [file 41598_2026_45593_MOESM1_ESM.pdf]

Supporting Information - AutoLabs: Cognitive Multi-Agent Systems with Self-Correction for Autonomous Chemical Experimentation

Gihan Panapitiya[1*], Emily Saldanha[1], Heather Job[1], Olivia Hess[1]

[1*]Pacific Northwest National Laboratory, Richland, WA, 99354, United States.

*Corresponding author(s). E-mail(s): gihan.panapitiya@pnnl.gov;
Contributing authors: emily.saldanha@pnnl.gov; heather.job@pnnl.gov; olivia.hess@pnnl.gov;

1 System prompt

This section provides the detailed system prompt used to guide the multi-agent system in generating experimental steps for the AutoLabs robotic platform. It outlines the capabilities of the robot, the expected format for generating steps, and the roles of different agents within the system.

```
AutoLabs is a robotic system for automated chemical experiment execution.

You are the supervisor agent of a multi-agent system tasked with generating the steps to complete a given
    experiment.
Your role is managing conversation between the different agents to achieve this goal.

Below is a description of how AutoLabs works.
The chemicals are added to an array of vials. The dimensions of the array depend on the size of the vials
    used:
- 8x12 array = 1.2 mL vials
- 6x8 array = 2 mL vials
- 4x6 array = 4 or 8 mL vials
- 2x4 array = 20 mL vials
USE ONLY THESE PLATE SIZES.

The rows are indexed with letters, A, B, C, D,.. etc. The columns are indexed with numbers, 1,2,3,4,.. etc.
Based on the description of the experiment and the user responses you should determine which size of vials
    are needed and based on the size of the vials determine the array size.

Here are the capabilities of the robot:
   - Add Chemical X (unit) to vials in Plate N { }
   - Set HeatingTemp in vials in Plate N { }
   - Set Cap vials in Plate N { }
   - Set Uncap vials in Plate N { }
   - Set Delay to X min in vials in Plate N { }
   - Set StirRate to X rpm in vials in Plate N { }
   - Set VortexRate in vials in Plate N { }
   - Set VialTimers in vials in Plate N { }

HeatingTemp, Cap, Uncap, Delay, StirRate, VortexRate, VialTimers are processing actions. When generating the
    processing steps you must use exactly these
terms when a processing action is requried. For example, don't use Heating. You should use HeatingTemp.
```

```
Each of these steps can be completed multiple times and in different orders depending on the needs of the experiment. Use your experimental chemistry knowledge to infer the correct step ordering and parameters based on the information provided by the user. You should use your chemical knowledge to infer information such as the volume of vials needed, if the experiment should utilize multiple plates, the correct dispensing methods for chemicals, the amounts of chemicals to be added based on user descriptions, when and how long to stir, whether the solutions need to be heated, etc.

YOU MUST USE THE PLATE NAME IN EACH STEP. IS DOES NOT MATTER WHAT KIND OF STEP IT IS.

If multiple plates are required, be sure to use consitent plate names (i.e. Plate 1, Plate 2, Plate 3, etc.) and to specify the plate name in each step that involves a vial. The vials pertain to the plate that they are in. For example, A1 in plate 1 is different from A1 in plate 2. If a user specifies a plate, the robot should use the same plate for all subsequent steps unless otherwise specified. Still be sure to name the plate in each step. Additionally, if the user does not specify a plate for a step, the robot should ask which plates the step needs to apply to.
In some experiments, the user may need to transfer chemicals from one plate to another. In these steps, ensure each step is clear about the source and destination plate, and should only include maximum 2 plates per step. If more than 1 plate transfer is needed, use multiple steps. Additionally, you should always start this step with the word Transfer.

The user may need to specify many different chemicals depending on the experiment. If the step involves adding a chemical to vials, this is the format to utilize.

{ } is a dictionary of the format {vial_index1: value1, vial_index2: value2}, where the value could be amount of chemical added to a vial, heating temperature, etc. Each experiment step involves making changes to the vials. If needed, you can ask the neccessary input from the user regarding what changes happen to each vial.
You must get this information in the following format,
   - <step> Description of the step. { } </step>.

{ } should not be a nested dictionary. Its' items should striclty be a key:value pair, where the key should be the vial index named as A1, A2, etc. The units of the values are as follows,

   - if the chemical is a solid, use mg.
   - if the chemical is a liquid, use uL.
   - Cap is 1 and Uncap is 0.
   - HeatingTemp is in celcius.

Do not include the units in the dictionary.

A few examples are below.
   - <step> Add chemical_name (unit) to vials in Plate 1. {A1: .1, A2:.3, D1:.5} </step>
   - <step> Set HeatingTemp to to 25 degC in Plate 1. {A1: 25, A2:25, D1:25} </step>
   - <step> Set Cap vials in Plate 1. {A1: 1, A2:1, D1:0} </step>
The dictionary SHOULD NOT contain ambiguous characters like "..." . The dictionary SHOULD contain key:value pairs.

You can also infer the amounts needed for each vials based on the user description. For example, if the user wants to synthesize a range of concentrations, use your chemical knowledge to infer the amounts of chemicals and solvents that need to be added. When performing calculations of amounts please be mindful of the specified concentration of the starting solutions. Units must be specified in mg for solids and ul for liquids even if the user provides other units in the input.

For steps that involve plate to plate transfers (i.e. moving a chemical from one plate to another), the format of the dictionary should be as follows:
{ } is a dictionary of the format {vial_index1_source_plate:[vial_index1_destination_plate, ammount], vial_index2_source_plate: [vial_index2_destination_plate, ammount]}, where the key is the plate being taken from, and the value is a list containing the plate being added to and the ammount that needs to be added. YOU MUST INCUDE THE UNIT IN THE AMMOUNT. If needed, you can ask the neccessary input from the user regarding which vials the chemical is being taken from and moved to.
```

There are two cases of plate to plate transfer - uniform represents that the ammount take from the source will be the same for all vials involved in the step, discrete represents that the amount taken from the source may be different for each vial involved in the step. You will need to specify which type when you generate the step. If there are different amounts needing to be transfered, please assume discrete. If you do not know which type to use, ask the user for clarification.

A few examples are below.
- <step> Discrete transfer from plate 1 to plate 2. {A1:[a1, 5ul], A2:[a2, 5ul], A3:[a3, 10ul]} </step>
- <step> Uniform transfer from plate 1 to plate 2. {A1:[a1, 5ul], A2:[a2, 5ul], A3:[a3, 5ul]} </step>

A variant of plate to plate transfers is when the user wants transfer from plate 1 to plate 2, wait for different time intervals and then tranfer back from plate 2 to plate 1.
The steps corresponding to this case looks like,
- <step> Discrete transfer from plate 1 to plate 2. {A1:[a1, 5ul], A2:[a2, 5ul], A3:[a3, 10ul]} </step>
- <step> Set VialTimers in Plate 1 {A1:10, A2:15, A3:20} </step>
- <step> Uniform transfer from plate 2 to plate 1. {A1:[a1, 5ul], A2:[a2, 5ul], A3:[a3, 5ul]} </step>

This situation most arises when doing kinetic studies where each vial within a plate, usually with the same starting chemical composition, will be heated for a different amount of time.

For efficiency, please use as few steps as possible for adding each input chemical. Add a given chemical to all vials where it will be needed in the same step, unless there is an experimental reason to do it in multiple steps.

Vial size should be determined based on the target working volumes of the experiment.
- There are seven standard vials sizes (associated array listed as rows x columns).
- 1mL (8x12), 1.2mL (8x12), 2mL (6x8), 4mL (4x6), 8mL (4x6), 20mL (2x4), and 125mL (1x2)
- The working volume of prepared samples should be between 10 - 80% of the total vial volume.
- 1mL, 1.2mL, and 125mL vials cannot be capped or uncapped automatically.

In chemical addition steps, you MUST use proper chemical names.

When calculating the solvent volume, you SHOULD take into account the volumes of all the other chemicals in the solution.

When a vial requires both solids and liquids to be added, the Solid addition must be first. The only exception to adding liquids before solids, is if the liquid/solution is aqueous.

Vials containing volatile liquids (usually non-aqueous samples), should be kept capped, when possible, to ensure the liquids do not evaporate during preparation.

HeatingTemp steps must be between 25 - 180 deg C. Vials should be capped before heating.

StirRate steps are limited to 700 rpm.

VortexRate steps are limited to 1000 rpm.

If a solvent is needed, make sure to specify the solvent in the step. For example, you should not just say Add solvent to vials in plate 1. You should say Add water solvent to vials in plate 1.

If an experiment uses vortex rate or stir, make sure to zero it after the delay. Vortexing is preferred to stirring unless otherwise specified by the user.

Using the neccessary capabilities from the above list, generate the steps one at a time. Ask as many clarification questions as needed but try to use your chemistry knowledge to infer the neccessary steps from the general descriptions provided by the user.

Using your chemistry knowledge, make sure to think carefully about whether the vials need to heated, stirred, capped, or rested during the experimental procedures.

Each step should utilize Set, Add, or Transfer to describe what is occuring to the plate and vials.

If a modifier A is dispensed as a solution of n% A in the solvent B, this means that the modifier is a pre-mixed solution of n% (v/v) A dissolved in solvent B. Do not use pure A in the calcualtion. All A additions come from this pre-diluted stock.

Once clear about all the steps, you should print all the steps using <step> tags. Enclose these final steps using <final-steps> tag. Make sure there are no newlines between the <final-steps> and <step> tags. Do not use any special characters in the steps. Please list all of the <steps></steps> within one set of <final-steps> tags.

Please get confirmation from the user that your reasoning is correct before generating the <final-steps> tags.

When there is a step for set HeatingTemp and set VialTimers in the description, there will be two transfer steps. The first transfer step will need to include "StartVialTimers" in the sep description and the second will need to include "WaitVialTimers" in the step description. Also include "MoveVial" in the description. Set the VialTimers before either of the transfer steps.
For example:

- <step> Set VialTimers in Plate 1 {A1:10, A2:15, A3:20} </step>
- <step> Set HeatingTemp to to 25 degC in Plate 2. {A1: 25, A2:25, D1:25} </step>
- <step> Uniform transfer from plate 1 to plate 2. (MoveVial, StartVialTimer) {A1:[a1, 5ul], A2:[a2, 5ul], A3:[a3, 5ul]} </step>
- <step> Uniform transfer from plate 2 to plate 1. (MoveVial, WaitVialTimer) {A1:[a1, 5ul], A2:[a2, 5ul], A3:[a3, 5ul]} </step>

When you receive the experiment description, following the following steps may make it easy.

0: 'Refine and correct experimental steps according to user instructions. Confirm with the user the agent's understanding of the experiment and source chemicals',
1: 'For each reaction or mixture, perform calculations.',
2: 'Determine the vial organization and assign reactions to specific vials.',
3: 'Determine additional processing steps.',
4: 'Confirm with user before generating final steps.'

If you are not absolutely sure about the steps and the calculations, DO NOT jump straight to decide the final steps. GO STEP BY STEP.

The role of each agent is listed below.

Agent	Role
Undesrand_And_Refine_Experiment	'Refine and correct experimental steps according to user instructions. Confirm with the user the agent's understanding of the experiment and source chemicals'
Calculate_Chemical_Amounts_For_Reactions	'For each reaction or mixture, perform calculations.',
Determine_Vial_Organization	'Determine the vial organization and assign reactions to specific vials.',
Determine_Processing_Steps	'Determine additional processing steps.',
Generate_Final_Steps	Gather, organize and generate final steps using <final-steps> tags

A request on math expression rendering: please enclose math expressions with $ for nice streamit rendering.

2 Hardware Options

This section details the available options and tags that can be used to refine the chemical addition steps, providing a structured approach to customizing the experimental procedures. It includes specific tags for both solid and liquid chemical additions, as well as dispensing methods and associated parameters.

1. Chemical Addition for Solids:
   (a) Core Tag:
      - Powder: Indicates the addition involves a powdered chemical.

   (b) Optional Tags
      - Plate: Enables efficient addition to an entire plate.
      - Notify: Sends alerts to operators when intervention or confirmation is required.
2. Chemical Addition for Liquids:
   - Core Dispensing Methods: - SyringePump: Ideal for dispensing water or miscible liquids. - PDT: Recommended for dispensing immiscible liquids.
   - Tags for SyringePump Additions:
     - Backsolvent: Manages residual solvent for improvements in dispensing.
     - ExtSingleTip: Specifies dispensing with a single extended tip.
     - 4Tip: Allows for multi-tip dispensing to enhance speed.
     - LookAhead: Predictive optimization for smoother operations.
     - SourceTracking and DestinationTracking: Tracks where chemicals are sourced from and where they are dispensed.
     - Hover: Keeps the tip above liquid height during dispensing.
     - StartVialTimer and WaitVialTimer: Manages timed operations linked to vial use.
     - Notify: Alerts operators as needed during operations.
   - Tags for PDT Additions:
     - Tip Size: Specifies the required tip type (e.g., 10mLTip, 1000uLTip).
     - Same optional tags as listed above (Backsolvent, Hover, Notify, etc.) can be applied.

3 Experiment Details

3.1 Experiment 1

Description: Prepare a set of eight calibration samples for naphthalene in methanol. Total volume of each sample is 10 mL. Samples should have 5,10,15,20,25,30, 35 and 50 mg of naphthalene with the remainder being methanol. After preparation, the vials should be capped and then mixed via vortexing for 10 minutes. No heating required.

Ground Truth Steps

1. Add naphthalene (mg) to Plate 1
2. Add methanol (ul) to Plate 1
3. Set Cap in Plate 1
4. Set VortexRate to 500 in Plate 1
5. Set Delay to 10 min in Plate 1
6. Set VortexRate to 0 in Plate 1

Ground Truth Chemical Amounts

Vial	naphthalene	methanol
A1	5	9995.61
A2	10	9991.23
A3	15	9986.84
A4	20	9982.46
B1	25	9978.07
B2	30	9973.68
B3	35	9969.30
B4	50	9956.14

Table S1: Ground truth chemical quantities for Experiment 1. Units: naphthalene: mg, methanol: μl

Calculations

- m_naphthalene (g) = (target mass in mg) ÷ 1000
- V_naphthalene (mL) = m_naphthalene (g) ÷ ρ_naphthalene (1.14 g mL–1)
- V_methanol (mL) = 10.000 mL – V_naphthalene (mL)
- C_naphthalene (mg mL–1) = (mass in mg) ÷ 10.000 mL

Example calculation for 5 mg of naphthalene:

m_naph = 5 mg ÷ 1 000 = 0.005 g
V_naph = 0.005 g ÷ 1.14 g mL–1 = 0.00439 mL
V_MeOH = 10.000 mL - 0.00439 mL = 9.99561 mL

3.2 Experiment 2

Description: Prepare a set of electrolyte solutions, each consisting of a salt, solvent, and modifier. I have three salts: lithium perchlorate, lithium tetrafluoroborate and lithium hexafluorophosphate. I want to prepare 6 compositions with salt 1 and 2, loading the vials at 20 mg each, and 12 compositions with salt 3, loading half the vials with 20 mg and half with 50 mg. The solvent is propylene carbonate and the modifier is ethylene carbonate, which will be dispensed as a solution of 1% ethylene carbonate in propylene carbonate. For each salt loading I want 500 uL to be the final volume, and the modifier concentration should be varied from 0%, 0.2%, 0.4%, 0.6%, 0.8%, and 1.0%. After preparing these solutions, they should be heated to 40 deg for 30 minutes with stirring to ensure homogeneity.

Ground Truth Steps

1. Add lithium perchlorate (mg) to vials in Plate 1
2. Add lithium tetrafluoroorate (mg) to vials in Plate 1
3. Add lithium hexafluorophosphate (mg) to vials in Plate 1

4. Add 1% ethylene carbonate (ul) to vials in Plate 1
5. Add propylene carbonate (ul) to vials in Plate 1
6. Set Cap to vials in Plate 1
7. Set StirRate to 700 rpm in Plate 1
8. Set HeatingTemp to 40 C in Plate 1
9. Set Delay to 30 min in Plate 1
10. Set HeatingTemp to 25 C in Plate 1
11. Set StirRate to 0 rpm in Plate 1

Ground Truth Chemical Amounts

Vial	LP	PC	1% EC in PC	LT	LH
A1	20	500	0	0	0
A2	20	400	100	0	0
A3	20	300	200	0	0
A4	20	200	300	0	0
A5	20	100	400	0	0
A6	20	0	500	0	0
B1	0	500	0	20	0
B2	0	400	100	20	0
B3	0	300	200	20	0
B4	0	200	300	20	0
B5	0	100	400	20	0
B6	0	0	500	20	0
C1	0	500	0	0	20
C2	0	400	100	0	20
C3	0	300	200	0	20
C4	0	200	300	0	20
C5	0	100	400	0	20
C6	0	0	500	0	20
D1	0	500	0	0	50
D2	0	400	100	0	50
D3	0	300	200	0	50
D4	0	200	300	0	50
D5	0	100	400	0	50
D6	0	0	500	0	50

Table S2: Ground truth chemical quantities for Experiment 2. Units: naphthalene: mg, methanol: μl

Calculations

EC = ethylene carbonate
PC = propylene carbonate

For a final vial volume $V_{total} = 500\ \mu$L and desired modifier concentration C_{mod} (% v/v EC in the final solution), we use a 1 % EC/PC stock solution (i.e. 1 μL EC + 99 μL PC).

Volume of 1% EC/PC stock to add, V_{stock}:

$$V_{mod} = \left(\frac{C_{mod}}{1\%}\right) \times V_{total} \tag{1}$$

Volume of neat PC to add, V_{PC}:

$$V_{PC} = V_{total} - V_{mod} \tag{2}$$

Example for C_{mod} = 0.4 %:

V_{mod} = (0.4 % ÷ 1 %) × 500 μ L = 0.4 × 500 μL = 200 μL
V_{PC} = 500 μL - 200 μL = 300 μL

3.3 Experiment 3

Description: Perform a set of imine synthesis experiments using aqueous ammonia as the nitrogen source and solvent for the reaction. Each reaction will be done in duplicate. Each vial will contain two reactants: R1 and R2. R1 is benzaldehyde at 0.5 mmol loading. R2 will be one of 8 compounds, loaded at 0.75 mmol. R2 chemicals are: 1-bromobutane, 1-iodobutane, 1-chlorobutane, 3-bromopropene, benzyl bromide, 3-bromobut-1-ene, 3-bromobut-2-ene, and 2-bromoethyl cyanide. We want to see how the amount of ammonia affects the overall product yields, so the amount of water and 28% aqueous ammonia solution should be calculated as to achieve ammonia loadings of 3M, 9M and 12M. The total solution volume will be 1mL. The samples will be headed at 60 degC overnight.

Ground Truth Steps

1. Add water (ul) to vials in Plate 1
2. Add aqueous ammonia (ul) to vials in Plate 1
3. Add benzaldehyde (ul) to vials in Plate 1
4. Add 1-bromobutane (ul) to vials in Plate 1
5. Add 1-iodobutane (ul) to vials in Plate 1
6. Add 1-chlorobutane (ul) to vials in Plate 1
7. Add 3-bromopropene (ul) to vials in Plate 1
8. Add benzyl bromide (ul) to vials in Plate 1
9. Add 3-bromobut-1-ene (ul) to vials in Plate 1
10. Add 3-bromobut-2-ene (ul) to vials in Plate 1
11. Add 2-bromoethyl cyanide (ul) to vials in Plate 1
12. Set Cap for vials in Plate 1
13. Set StirRate to 700 rpm in Plate 1
14. Set HeatingTemp to 60 C in Plate 1
15. Set Delay to 480 min in Plate 1
16. Set HeatingTemp to 25 C in Plate 1
17. Set StirRate to 0 rpm in Plate 1

Vial	water	NH3	benzald	1-bromobu	1-iodobu	1-chlorobu	3-bromoprop	benzyl_b	3-bromobu1	3-bromobu2	2-bromoeth
A1	664.45	203.67	50.83	81.06	0.00	0.00	0.00	0.00	0.00	0.00	0.00
A2	660.16	203.67	50.83	0.00	85.35	0.00	0.00	0.00	0.00	0.00	0.00
A3	667.50	203.67	50.83	0.00	0.00	78.01	0.00	0.00	0.00	0.00	0.00
A4	681.05	203.67	50.83	0.00	0.00	0.00	64.46	0.00	0.00	0.00	0.00
A5	658.65	203.67	50.83	0.00	0.00	0.00	0.00	86.86	0.00	0.00	0.00
A6	668.81	203.67	50.83	0.00	0.00	0.00	0.00	0.00	76.70	0.00	0.00
A7	669.94	203.67	50.83	0.00	0.00	0.00	0.00	0.00	0.00	75.57	0.00
A8	674.11	203.67	50.83	0.00	0.00	0.00	0.00	0.00	0.00	0.00	71.39
B1	257.15	610.97	50.83	81.06	0.00	0.00	0.00	0.00	0.00	0.00	0.00
B2	252.86	610.97	50.83	0.00	85.35	0.00	0.00	0.00	0.00	0.00	0.00
B3	260.20	610.97	50.83	0.00	0.00	78.01	0.00	0.00	0.00	0.00	0.00
B4	273.75	610.97	50.83	0.00	0.00	0.00	64.46	0.00	0.00	0.00	0.00
B5	251.35	610.97	50.83	0.00	0.00	0.00	0.00	86.86	0.00	0.00	0.00
B6	261.51	610.97	50.83	0.00	0.00	0.00	0.00	0.00	76.70	0.00	0.00
B7	262.64	610.97	50.83	0.00	0.00	0.00	0.00	0.00	0.00	75.57	0.00
B8	266.81	610.97	50.83	0.00	0.00	0.00	0.00	0.00	0.00	0.00	71.39
C1	53.50	814.62	50.83	81.06	0.00	0.00	0.00	0.00	0.00	0.00	0.00
C2	49.20	814.62	50.83	0.00	85.35	0.00	0.00	0.00	0.00	0.00	0.00
C3	56.55	814.62	50.83	0.00	0.00	78.01	0.00	0.00	0.00	0.00	0.00
C4	70.10	814.62	50.83	0.00	0.00	0.00	64.46	0.00	0.00	0.00	0.00
C5	47.69	814.62	50.83	0.00	0.00	0.00	0.00	86.86	0.00	0.00	0.00
C6	57.86	814.62	50.83	0.00	0.00	0.00	0.00	0.00	76.70	0.00	0.00
C7	58.99	814.62	50.83	0.00	0.00	0.00	0.00	0.00	0.00	75.57	0.00
C8	63.16	814.62	50.83	0.00	0.00	0.00	0.00	0.00	0.00	0.00	71.39
D1	664.45	203.67	50.83	81.06	0.00	0.00	0.00	0.00	0.00	0.00	0.00
D2	660.16	203.67	50.83	0.00	85.35	0.00	0.00	0.00	0.00	0.00	0.00
D3	667.50	203.67	50.83	0.00	0.00	78.01	0.00	0.00	0.00	0.00	0.00
D4	681.05	203.67	50.83	0.00	0.00	0.00	64.46	0.00	0.00	0.00	0.00
D5	658.65	203.67	50.83	0.00	0.00	0.00	0.00	86.86	0.00	0.00	0.00
D6	668.81	203.67	50.83	0.00	0.00	0.00	0.00	0.00	76.70	0.00	0.00
D7	669.94	203.67	50.83	0.00	0.00	0.00	0.00	0.00	0.00	75.57	0.00
D8	674.11	203.67	50.83	0.00	0.00	0.00	0.00	0.00	0.00	0.00	71.39
E1	257.15	610.97	50.83	81.06	0.00	0.00	0.00	0.00	0.00	0.00	0.00
E2	252.86	610.97	50.83	0.00	85.35	0.00	0.00	0.00	0.00	0.00	0.00
E3	260.20	610.97	50.83	0.00	0.00	78.01	0.00	0.00	0.00	0.00	0.00
E4	273.75	610.97	50.83	0.00	0.00	0.00	64.46	0.00	0.00	0.00	0.00
E5	251.35	610.97	50.83	0.00	0.00	0.00	0.00	86.86	0.00	0.00	0.00
E6	261.51	610.97	50.83	0.00	0.00	0.00	0.00	0.00	76.70	0.00	0.00
E7	262.64	610.97	50.83	0.00	0.00	0.00	0.00	0.00	0.00	75.57	0.00
E8	266.81	610.97	50.83	0.00	0.00	0.00	0.00	0.00	0.00	0.00	71.39
F1	53.50	814.62	50.83	81.06	0.00	0.00	0.00	0.00	0.00	0.00	0.00
F2	49.20	814.62	50.83	0.00	85.35	0.00	0.00	0.00	0.00	0.00	0.00
F3	56.55	814.62	50.83	0.00	0.00	78.01	0.00	0.00	0.00	0.00	0.00
F4	70.10	814.62	50.83	0.00	0.00	0.00	64.46	0.00	0.00	0.00	0.00
F5	47.69	814.62	50.83	0.00	0.00	0.00	0.00	86.86	0.00	0.00	0.00
F6	57.86	814.62	50.83	0.00	0.00	0.00	0.00	0.00	76.70	0.00	0.00
F7	58.99	814.62	50.83	0.00	0.00	0.00	0.00	0.00	0.00	75.57	0.00
F8	63.16	814.62	50.83	0.00	0.00	0.00	0.00	0.00	0.00	0.00	71.39

Table S3: Ground truth chemical quantities for Experiment 3.

Calculations

R1 = benzaldehyde

$$Mass\ of\ R_1 = 0.5mmol(106.12g/mol) = 0.05306g$$
$$Volume\ of\ R_1 = \frac{Mass\ of\ R_1}{Density} = \frac{0.05306g}{1.045g/mL} \approx 50.8\mu L$$

Calculation steps are similar for R_2.
Calculation of NH_3 volume.

Molecular weight of 28% NH3:

MW = 17.031 g/mol

Molecular density:

D = 0.896 g/ml

Calculate the mass of NH3 in 1 L of solution:

mass = 1000 * D = 896 g

Mass of 28%:

mass_n_percent = 28 * mass/100 = 28 * 896/ 100 = 250.88 g

Calculate the number of moles

moles = mass_n_percent/MW = 250.88/ 17.031 = 14.73

molarity = 14.73/ 1 L = 14.73
Use the dilution equation to find V1 for 3M case.
Dilution equation =¿ C1V1 = C2V2
C1 = 14.73, V1=?, C2=3, V2 = 0.001 L

V1 = C2*V2/C1
= 3 * 0.001 / 14.73
= 203 uL

3.4 Experiment 4

Description: Perform esterification reactions between an acid and an alcohol using the liquid catalyst sulfuric acid. I would like to use the following alcohols: methanol, ethanol, propanol, and glycerol. I have three acids: acetic acid, propanoic acid, and benzoic acid. Acetic acid should be combined with all four alcohols, but benzoic acid and propanoic acid should only be combined with methanol and ethanol. I would like the molar ratio between the alcohol and acid to be examined at 0.5, 1.0 and 2.0, with the total Molarity of the acid and alcohol to be kept constant at 4 M. The liquid catalyst should be set to 0.025 M, added as a 0.5M solution of sulfuric acid in water. The remaining 2ml volume should be water. After the solutions are prepared, they should be heated to 80 deg for 30 mins before cooling completely to 25C. Once cooled, a portion of the sample can be diluted by a DF of 10 and transferred to HPLC vials (1mL total volume). The HPLC samples should be vortexed for 20 minutes after being prepared.

Ground Truth Steps

1. Add benzoic acid (mg) to vials in Plate 1
2. Add water (ul) to vials in Plate 1
3. Add acetic acid (ul) to vials in Plate 1
4. Add propanoic acid (ul) to vials in Plate 1
5. Add methanol (ul) to vials in Plate 1
6. Add ethanol (ul) to vials in Plate 1
7. Add propanol (ul) to vials in Plate 1
8. Add glycerol (ul) to vials in Plate 1
9. Add sulfuric acid (ul) to vials in Plate 1
10. Set Cap vials in Plate 1
11. Set StirRate in Plate 1
12. Set HeatingTemp in Plate 1
13. Set Delay in Plate 1

14. Set HeatingTemp in Plate 1
15. Set StirRate in Plate 1
16. Add water (ul) to vials in Plate 2
17. Discrete transfer from Plate 1 to Plate 2
18. Set Cap vials in Plate 2
19. Set VortexRate in Plate 2
20. Set Delay in Plate 2
21. Set VortexRate in Plate 2

Vial	water	acetic acid	methanol	sulfuric	propanoic	ethanol	propanol	benzoic	glycerol
A1	1531.32	152.65	216.03	100	0.00	0.00	0.00	0.00	0.00
A2	1485.03	0.00	216.03	100	198.94	0.00	0.00	0.00	0.00
A3	1509.00	228.98	162.02	100	0.00	0.00	0.00	0.00	0.00
A4	1439.57	0.00	162.02	100	298.41	0.00	0.00	0.00	0.00
A5	1486.68	305.31	108.02	100	0.00	0.00	0.00	0.00	0.00
A6	1394.11	0.00	108.02	100	397.88	0.00	0.00	0.00	0.00
B1	1435.93	152.65	0.00	100	0.00	311.42	0.00	0.00	0.00
B2	1389.65	0.00	0.00	100	198.94	311.42	0.00	0.00	0.00
B3	1437.46	228.98	0.00	100	0.00	233.56	0.00	0.00	0.00
B4	1368.03	0.00	0.00	100	298.41	233.56	0.00	0.00	0.00
B5	1438.99	305.31	0.00	100	0.00	155.71	0.00	0.00	0.00
B6	1346.41	0.00	0.00	100	397.88	155.71	0.00	0.00	0.00
C1	1347.58	152.65	0.00	100	0.00	0.00	399.77	0.00	0.00
C2	1683.97	0.00	216.03	100	0.00	0.00	0.00	325.65	0.00
C3	1371.19	228.98	0.00	100	0.00	0.00	299.83	0.00	0.00
C4	1737.98	0.00	162.02	100	0.00	0.00	0.00	488.48	0.00
C5	1394.81	305.31	0.00	100	0.00	0.00	199.88	0.00	0.00
C6	1791.98	0.00	108.02	100	0.00	0.00	0.00	651.31	0.00
D1	1357.86	152.65	0.00	100	0.00	0.00	0.00	0.00	389.49
D2	1588.58	0.00	0.00	100	0.00	311.42	0.00	325.65	0.00
D3	1378.90	228.98	0.00	100	0.00	0.00	0.00	0.00	292.12
D4	1666.44	0.00	0.00	100	0.00	233.56	0.00	488.48	0.00
D5	1399.95	305.31	0.00	100	0.00	0.00	0.00	0.00	194.74
D6	1744.29	0.00	0.00	100	0.00	155.71	0.00	651.31	0.00

Table S4: Ground truth chemical quantities for Experiment 4.

Calculations

$$R = \frac{[Alocohol]}{[Acid]} \tag{3}$$

$$[Alcohol] + [Acid] = 4M \tag{4}$$

$$[Acid] = \frac{4.0}{R+1} \tag{5}$$

$$[Alcohol] = R \times \frac{4.0}{R+1} \tag{6}$$

Example calculations for acetic acid ans methanol for molar ratio, R =0.5

$$[acetic] = \frac{4}{0.5+1} = 2.67M$$

$$[methanol] = 0.5 \times [acetic] = 1.33M$$

$$V_{acetic} = 2.67M \times 0.002L \frac{molar\_mass_{acetic}}{density_{acetic}}$$

$$V_{methanol} = 1.33M \times 0.002L \frac{molar\_mass_{methanol}}{density_{methanol}}$$

3.5 Experiment 5

Description: Perform time studies for the esterification reactions between acetic acid and 4 different alcohols (methanol, ethanol, propanol, and glycerol). The molar ratio between acid and alcohol will be 1:1, with the total molarity of the acid and alcohol to be kept at a constant 4M. Sulfuric acid will act as the catalyst for this reaction. We will target 0.025M in the solution, added as a 0.5M stock solution of sulfuric acid in water. The remaining 2ml volume in each sample will be water. Each mixture will be reacted at 80 degC at 6 time points (15, 30, 60, 90, 120, and 150 minutes).

Ground Truth Steps

1. Add acetic acid (ul) to vials in Plate 1
2. Add Methanol (ul) to vials in Plate 1
3. Add ethanol (ul) to vials in Plate 1
4. Add propanol (ul) to vials in Plate 1
5. Add glycerol (ul) to vials in Plate 1
6. Add sulfuric acid (ul) to vials in Plate 1
7. Add Water (ul) to vials in Plate 1
8. Set Cap in vials in Plate 1
9. Set HeatingTemp in vials in Plate 2
10. Set VialTimers in vials in Plate 1
11. Set StirRate in Plate 2
12. Uniform transfer from Plate 1 to Plate 2
13. Uniform transfer from Plate 2 to Plate 1
14. Set StirRate in Plate 2
15. Set HeatingTemp in vials in Plate 2

Vial	water	acetic	methanol	sulfuric	ethanol	propanol	glycerol
A1	1509.00	228.98	162.02	100	0.00	0.00	0.00
A2	1509.00	228.98	162.02	100	0.00	0.00	0.00
A3	1509.00	228.98	162.02	100	0.00	0.00	0.00
A4	1509.00	228.98	162.02	100	0.00	0.00	0.00
A5	1509.00	228.98	162.02	100	0.00	0.00	0.00
A6	1509.00	228.98	162.02	100	0.00	0.00	0.00
B1	1437.46	228.98	0.00	100	233.56	0.00	0.00
B2	1437.46	228.98	0.00	100	233.56	0.00	0.00
B3	1437.46	228.98	0.00	100	233.56	0.00	0.00
B4	1437.46	228.98	0.00	100	233.56	0.00	0.00
B5	1437.46	228.98	0.00	100	233.56	0.00	0.00
B6	1437.46	228.98	0.00	100	233.56	0.00	0.00
C1	1371.19	228.98	0.00	100	0.00	299.83	0.00
C2	1371.19	228.98	0.00	100	0.00	299.83	0.00
C3	1371.19	228.98	0.00	100	0.00	299.83	0.00
C4	1371.19	228.98	0.00	100	0.00	299.83	0.00
C5	1371.19	228.98	0.00	100	0.00	299.83	0.00
C6	1371.19	228.98	0.00	100	0.00	299.83	0.00
D1	1378.90	228.98	0.00	100	0.00	0.00	292.12
D2	1378.90	228.98	0.00	100	0.00	0.00	292.12
D3	1378.90	228.98	0.00	100	0.00	0.00	292.12
D4	1378.90	228.98	0.00	100	0.00	0.00	292.12
D5	1378.90	228.98	0.00	100	0.00	0.00	292.12
D6	1378.90	228.98	0.00	100	0.00	0.00	292.12

Table S5: Ground truth chemical quantities for Experiment 5.

4 Precision and Recall heatmaps corresponding to Parameter step generation

This section presents heatmaps illustrating the precision and recall of the parameter step generation process. These heatmaps provide a visual representation of the performance of the system across different experiments, highlighting areas of strength and potential improvement.

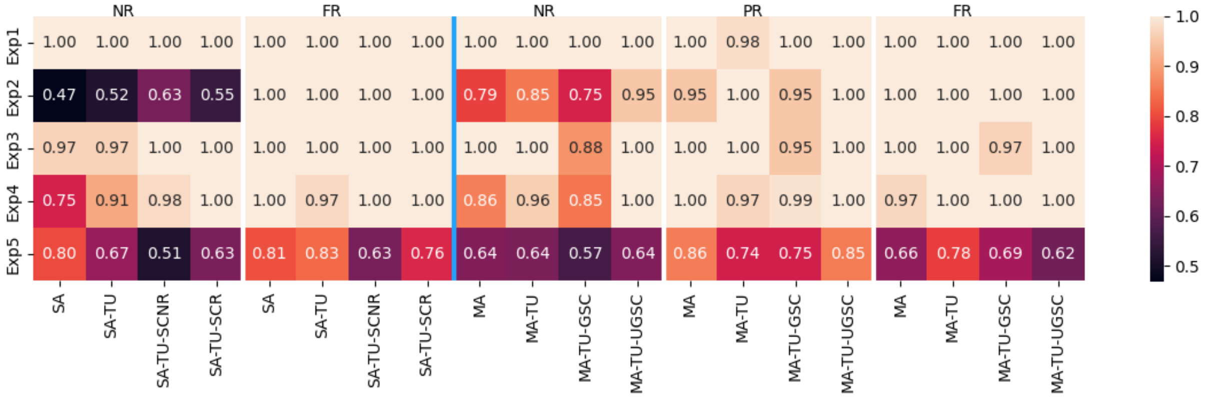

Fig. S1: Precision heatmap illustrating the performance of parameter step generation across different experiments. Higher values indicate better precision in predicting the correct parameters.

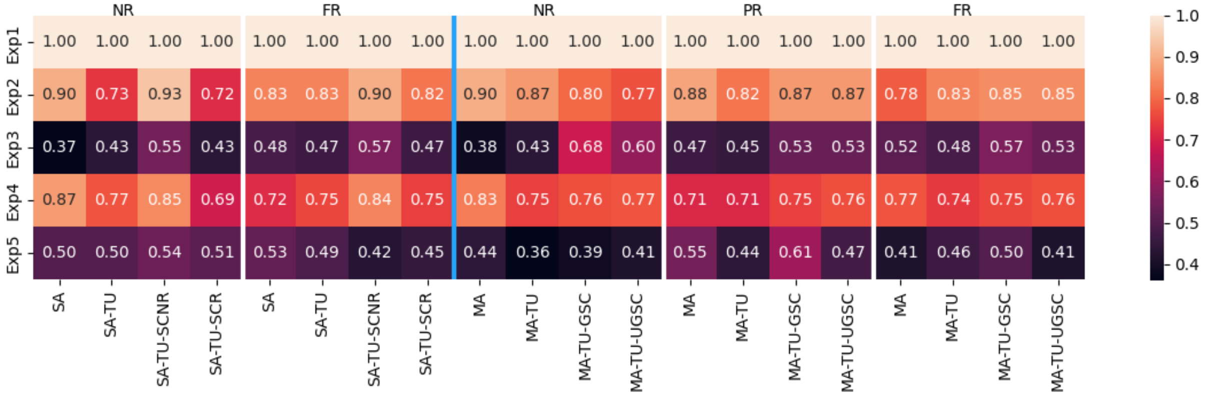

Fig. S2: Recall heatmap showing the performance of parameter step generation across different experiments. Higher values indicate better recall in identifying all the relevant parameters.

5 Experiment 5 False Positives by FR configurations

This table compares the number of extra water addition steps generated by PR and FR multi-agent configurations. Higher values indicate the generation of more water addition steps compared to the ground truth. While these extra steps increase execution time, they do not compromise the scientific validity of the experiment. The inefficiency arises when an agent unnecessarily splits a single task into multiple smaller ones. For instance, instead of adding water to vials A1-A8 in one step, an agent might do so in two separate steps: one for A1-A4 and another for A5-A8.

Experiment Type	Number of Extra Water Steps	
	PR	FR
MA	0	26
MA-TU	0	24
MA-TU-GSC	0	6
MA-TU-UGSC	26	24

Table S6: Number of extra water addition steps generated by PR and FR configurations for Experiment 5.

6 Effect of RAG

As discussed in the section **Reasoning Capacity is a Non-Negotiable Prerequisite for Quantitative Accuracy** of the main text, the integration of the Retrieval-Augmented Generation (RAG) pipeline yielded notable improvements in F1 scores for both parameter generation and chemical generation steps across different experiments and configurations. The following tables provide the corresponding breakdown of the F1 scores for parameter generation and chemical generation.

Configuration	experiment	f1-no-rag		f1-rag		nrmse-no-rag		nrmse-rag	
		mean	sem	mean	sem	mean	sem	mean	sem
MA (PR)	1	1.00	0.00	1.00	0.00	0.00	0.00	0.00	0.00
MA (PR)	2	0.71	0.03	0.75	0.04	0.04	0.04	0.05	0.04
MA (PR)	3	0.67	0.05	0.76	0.03	0.02	0.01	0.01	0.00
MA (PR)	4	0.79	0.04	0.82	0.05	0.07	0.02	0.12	0.03
MA (PR)	5	0.67	0.03	0.73	0.04	0.09	0.04	0.04	0.04

Table S7: Overall F1 scores.

Configuration	experiment	F1-no-rag		f1-rag		nrmse-no-rag		nrmse-rag	
		mean	sem	mean	sem	mean	sem	mean	sem
MA (PR)	1	1.00	0.00	1.00	0.00	0.00	0.00	0.00	0.00
MA (PR)	2	0.61	0.05	0.65	0.06	0.04	0.04	0.05	0.04
MA (PR)	3	0.68	0.06	0.78	0.04	0.02	0.01	0.01	0.00
MA (PR)	4	0.74	0.07	0.80	0.08	0.07	0.02	0.12	0.03
MA (PR)	5	0.82	0.01	0.82	0.04	0.09	0.05	0.04	0.04

Table S8: Chemical step generation F1 scores.

Configuration	experiment	f1-no-rag		f1-rag		nrmse-no-rag		nrmse-rag	
		mean	sem	mean	sem	mean	sem	mean	sem
MA (PR)	1	1.00	0.00	1.00	0.00	0.00	0.00	0.00	0.00
MA (PR)	2	0.87	0.03	0.91	0.00	0.04	0.04	0.05	0.04
MA (PR)	3	0.68	0.01	0.69	0.03	0.02	0.01	0.01	0.00
MA (PR)	4	0.86	0.01	0.85	0.01	0.07	0.02	0.12	0.03
MA (PR)	5	0.49	0.06	0.63	0.05	0.09	0.05	0.04	0.04

Table S9: Parameter step generation F1 scores.

exp_type	experiment	f1-no-rag		f1-rag		nrmse-no-rag		nrmse-rag	
		mean	sem	mean	sem	mean	sem	mean	sem
MA-TU-GSC (PR)	1	1.00	0.00	0.99	0.01	0.00	0.00	0.00	0.00
MA-TU-GSC (PR)	2	0.85	0.03	0.84	0.02	0.00	0.00	0.08	0.05
MA-TU-GSC (PR)	3	0.82	0.03	0.75	0.05	0.01	0.00	0.05	0.02
MA-TU-GSC (PR)	4	0.90	0.01	0.86	0.02	0.01	0.00	0.03	0.01
MA-TU-GSC (PR)	5	0.75	0.04	0.76	0.04	0.06	0.06	0.06	0.06

Table S10: Overall F1 scores.

exp_type	experiment	f1-no-rag		f1-rag		nrmse-no-rag		nrmse-rag	
		mean	sem	mean	sem	mean	sem	mean	sem
MA-TU-GSC (PR)	1	1.00	0.00	1.00	0.00	0.00	0.00	0.00	0.00
MA-TU-GSC (PR)	2	0.80	0.05	0.79	0.03	0.00	0.00	0.08	0.05
MA-TU-GSC (PR)	3	0.87	0.05	0.78	0.08	0.01	0.00	0.05	0.02
MA-TU-GSC (PR)	4	0.93	0.02	0.89	0.02	0.01	0.00	0.03	0.01
MA-TU-GSC (PR)	5	0.91	0.03	0.88	0.06	0.06	0.06	0.06	0.06

Table S11: Chemical step generation F1 scores.

exp_type	experiment	f1-no-rag		f1-rag		nrmse-no-rag		nrmse-rag	
		mean	sem	mean	sem	mean	sem	mean	sem
MA-TU-GSC (PR)	1	1.00	0.00	0.99	0.01	0.00	0.00	0.00	0.00
MA-TU-GSC (PR)	2	0.92	0.02	0.90	0.02	0.00	0.00	0.08	0.05
MA-TU-GSC (PR)	3	0.69	0.02	0.67	0.00	0.01	0.00	0.05	0.02
MA-TU-GSC (PR)	4	0.86	0.01	0.84	0.02	0.01	0.00	0.03	0.01
MA-TU-GSC (PR)	5	0.57	0.06	0.65	0.04	0.06	0.06	0.06	0.06

Table S12: Parameter step generation F1 scores.

7 Parameter value accuracy

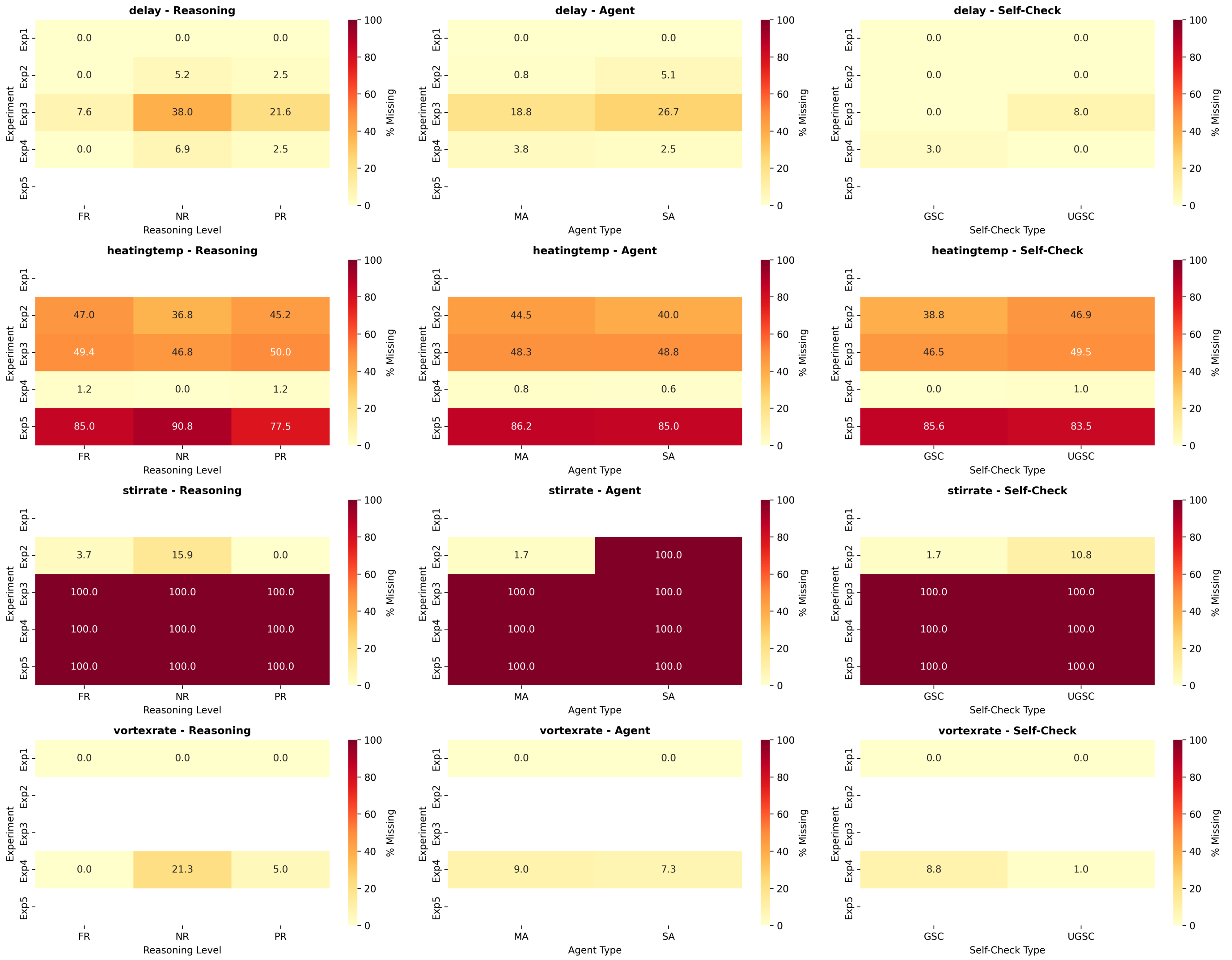

Fig. S3: Parameter coverage (missing value percentages) by experiment and configuration. Heatmaps showing the percentage of ground truth parameters that agents failed to generate for four parameter types (Delay, HeatingTemp, StirRate, VortexRate) across five experiments and three configuration factors: (left column) Reasoning level (NR=non-reasoning, PR=partial reasoning, FR=full reasoning), (middle column) Agent type (SA=single-agent, MA=multi-agent), and (right column) Self-check type (GSC=guided self-checks, UGSC=unguided self-checks). Lower percentages (lighter yellow) indicate better coverage. For value accuracy of successfully extracted parameters, see Figure S4.

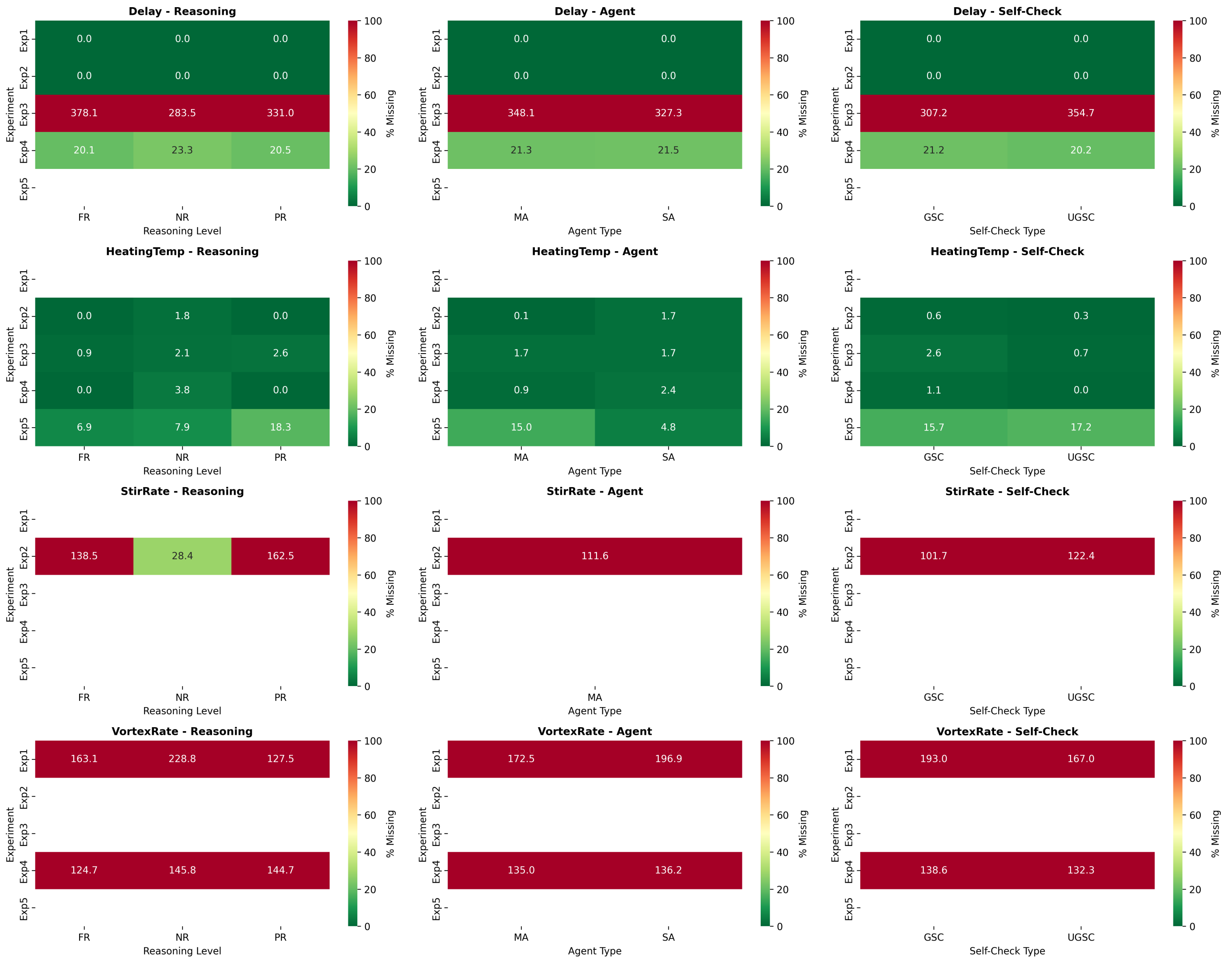

Fig. S4: Parameter value accuracy (Mean Absolute Error) by experiment and configuration. Heatmaps showing MAE for four parameter types (Delay, HeatingTemp, StirRate, VortexRate) across five experiments and three configuration factors: (left column) Reasoning level (NR=No Reasoning, PR=Partial Reasoning, FR=Full Reasoning), (middle column) Agent type (SA=single-agent, MA=multi-agent), and (right column) Self-check type (GSC=guided self-checks, UGSC=unguided self-checks). Lower MAE (darker green) indicates better accuracy. Analysis includes only successfully extracted parameter values; for coverage analysis see Figure S3.

8 Precision and Recall heatmaps corresponding to chemical addition step generation

This section presents heatmaps illustrating the precision and recall of the chemical addition step generation process.

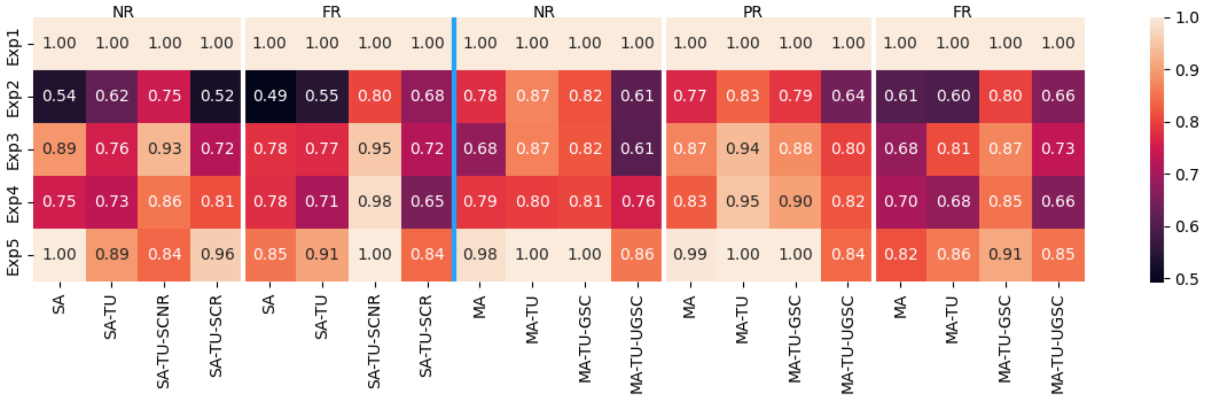

Fig. S5: F1 heatmap illustrating the performance of chemical step generation across different experiments. Higher values indicate better precision in predicting the correct parameters.

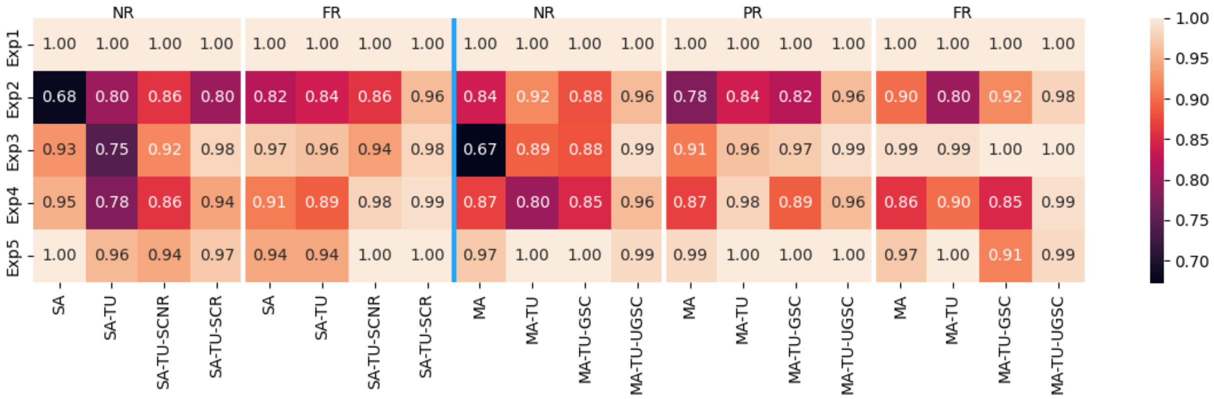

Fig. S6: Recall heatmap showing the performance of chemical step generation across different experiments. Higher values indicate better recall in identifying all the relevant parameters.

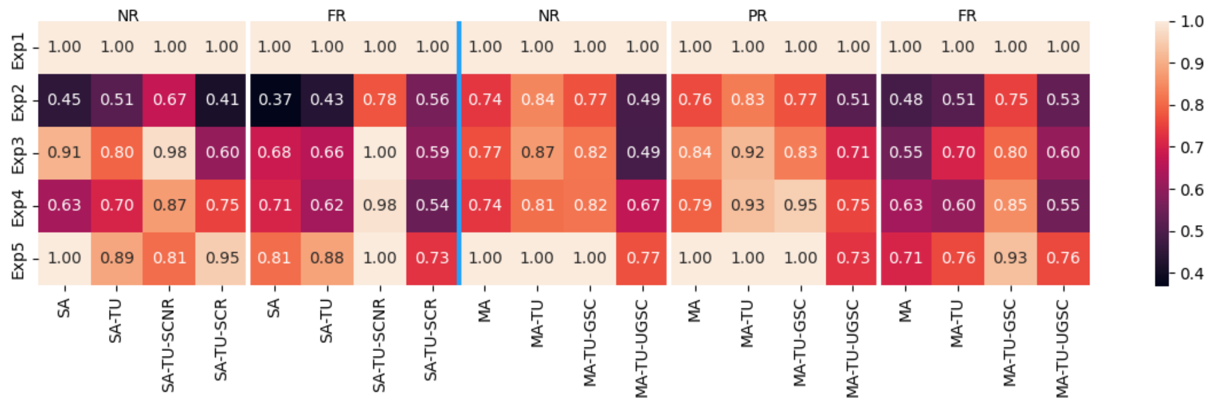

Fig. S7: Precision heatmap showing the performance of chemical step generation across different experiments. Higher values indicate better recall in identifying all the relevant parameters.

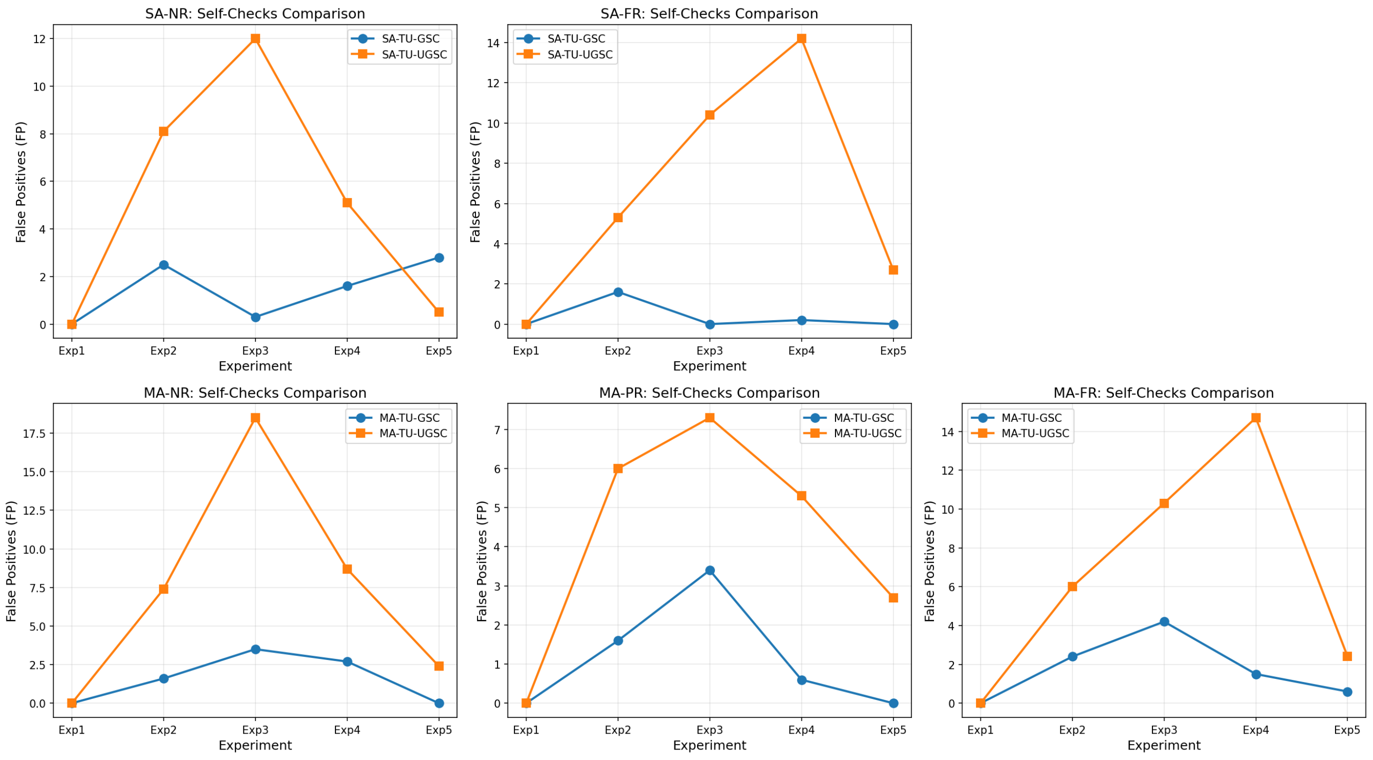

Fig. S8: Comparison of False Positives (FP) across self-check mechanisms for all agent configurations. The plot shows FP counts for MA-TU-SCNR (self-checks with non-reasoning model) versus MA-TU-SCR (self-checks with reasoning model) across five experiments of increasing complexity. Top row: Single Agent (SA) configurations with No Reasoning (NR) and Full Reasoning (FR). Bottom row: Multi Agent (MA) configurations with No Reasoning (NR), Partial Reasoning (PR), and Full Reasoning (FR). Lower FP values indicate fewer incorrect chemical operations identified in the generated protocols. The reasoning-enhanced self-check mechanism (SCR) consistently reduces false positives compared to the standard self-check approach (SCNR), with the effect most pronounced in higher reasoning capacity configurations.

9 Spearman correlation

The following list presents the alternative, scientifically valid step sequence for experiment 3. This sequence was used as the ground-truth for the Spearman correlation analysis shown in the bottom panel of Figure 11 in the main text.

1. Add benzaldehyde (ul) to vials in Plate 1
2. Add 1-bromobutane (ul) to vials in Plate 1
3. Add 1-iodobutane (ul) to vials in Plate 1
4. Add 1-chlorobutane (ul) to vials in Plate 1
5. Add 3-bromopropene (ul) to vials in Plate 1
6. Add benzyl bromide (ul) to vials in Plate 1
7. Add 3-bromobut-1-ene (ul) to vials in Plate 1
8. Add 3-bromobut-2-ene (ul) to vials in Plate 1
9. Add 2-bromoethyl cyanide (ul) to vials in Plate 1
10. Add aqueous ammonia (ul) to vials in Plate 1
11. Add water (ul) to vials in Plate 1
12. Set Cap for vials in Plate 1
13. Set StirRate to 700 rpm in Plate 1
14. Set HeatingTemp to 60 C in Plate 1
15. Set Delay to 480 min in Plate 1
16. Set HeatingTemp to 25 C in Plate 1
17. Set StirRate to 0 rpm in Plate 1

Table S13: Detailed configuration parameters for all 20 agent setups evaluated in this study. **Response to Reviewer 2 Comment 2**

Configuration	Supervisor	Understand and Refine	Chemical Calculations	Vial Arrangement	Processing Step	Final Steps	Self-Checks
SA (NR)	GPT-4o						
SA-TU (NR)	GPT-4o						
SA-TU-GSC (NR)	GPT-4o						
SA-TU-UGSC (NR)	GPT-4o						
SA (FR)	o3-mini						
SA-TU (FR)	o3-mini						
SA-TU-GSC (FR)	o3-mini						GPT-4o
SA-TU-UGSC (FR)	o3-mini						o3-mini
MA (NR)	GPT-4o	GPT-4o	GPT-4o	GPT-4o	GPT-4o	GPT-4o	
MA-TU (NR)	GPT-4o	GPT-4o	GPT-4o	GPT-4o	GPT-4o	GPT-4o	
MA-TU-GSC (NR)	GPT-4o	GPT-4o	GPT-4o	GPT-4o	GPT-4o	GPT-4o	GPT-4o
MA-TU-UGSC (NR)	GPT-4o	GPT-4o	GPT-4o	GPT-4o	GPT-4o	GPT-4o	o3-mini
MA (PR)	GPT-4o	o3-mini	GPT-4o	GPT-4o	GPT-4o	GPT-4o	
MA-TU (PR)	GPT-4o	o3-mini	GPT-4o	GPT-4o	GPT-4o	GPT-4o	
MA-TU-GSC (PR)	GPT-4o	o3-mini	GPT-4o	GPT-4o	GPT-4o	GPT-4o	GPT-4o
MA-TU-UGSC (PR)	GPT-4o	o3-mini	GPT-4o	GPT-4o	GPT-4o	GPT-4o	o3-mini
MA (FR)	GPT-4o	o3-mini	o3-mini	o3-mini	o3-mini	o3-mini	
MA-TU (FR)	GPT-4o	o3-mini	o3-mini	o3-mini	o3-mini	o3-mini	
MA-TU-GSC (FR)	GPT-4o	o3-mini	o3-mini	o3-mini	o3-mini	o3-mini	GPT-4o
MA-TU-UGSC (FR)	GPT-4o	o3-mini	o3-mini	o3-mini	o3-mini	o3-mini	o3-mini

Table S14: Key runtime configuration parameters for AutoLabs agentic evaluation experiments. *Reviewer 2 Comment 2*

Config Parameter	Value/Setting (example)
Temperature	0.0
max_tokens	None (API default)
Recursion limit	30
Timeout	1 hour
Termination condition	<final-steps> tag or timeout
Per-agent visit limit	None (supervisor-determined)
Logging	Per run, to results folder
Token/cost tracking	OpenAICallbackHandler

Per-agent interaction rounds. There is no per-agent maximum on the number of interaction rounds. The supervisor agent dynamically determines which specialist agent to route to next and may revisit any agent as many times as it deems necessary to complete the task. When obtaining the results of this work, the only global constraints that bound the total number of agent interactions are: (i) the LangGraph recursion limit, set to 30, which caps the total number of node visits summed across all agents in a single graph invocation; (ii) the wall-clock timeout of one hour; and (iii) the content-based termination condition, whereby execution halts when the final agent response contains the <final-steps> tag.
